# Ankyloblepharon–Ectodermal Defects–Cleft Lip/Palate Syndrome-Linked p63 Mutations Disrupt Keratinocyte Proliferation and Survival Through Oxidative Stress and Impaired Slc7a11 Expression

**DOI:** 10.3390/ijms26115231

**Published:** 2025-05-29

**Authors:** Daniela Di Girolamo, Sara Palumbo, Dario Antonini, Ludovica D’Auria, Vincenza Cerbone, Tommaso Porcelli, Federica Cavallo, Enzo Calautti, Chiara Riganti, Caterina Missero

**Affiliations:** 1 Department of Biology, University of Naples Federico II, 80126 Naples, Italy; dario.antonini@unina.it; 2 CEINGE Biotecnologie Avanzate Franco Salvatore, 80145 Naples, Italy; palumbos@ceinge.unina.it (S.P.); daurial@ceinge.unina.it (L.D.); cerbone@ceinge.unina.it (V.C.); 3 Department of Molecular Medicine and Medical Biotechnologies, University of Naples Federico II, 80131 Naples, Italy; 4 Department of Public Health, University of Naples Federico II, 80131 Napoli, Italy; tommaso.porcelli@unina.it; 5 Department of Molecular Biotechnology and Health Sciences, University of Turin, 10126 Turin, Italy; federica.cavallo@unito.it (F.C.); vincenzo.calautti@unito.it (E.C.); 6 Molecular Biotechnology Center “Guido Tarone”, University of Turin, 10126 Turin, Italy; chiara.riganti@unito.it; 7 Department of Oncology, University of Turin, 10126 Turin, Italy

**Keywords:** AEC syndrome, p63 protein, antioxidant defense, oxidative stress, keratinocyte proliferation, cell death

## Abstract

Mutations in the TP63 gene cause several syndromic disorders, including ankyloblepharon–ectodermal defects–cleft lip/palate (AEC) syndrome, characterized by severe skin erosions, cleft palate, and ectodermal dysplasia. These mutations often affect the carboxy-terminal sterile-α-motif (SAM) domain of the p63 protein, leading to domain misfolding, protein aggregation, and impaired transcriptional activity. To dissect the molecular mechanisms underlying AEC pathogenesis, we investigated primary keratinocytes derived from p63L514F mutant mice, which carry a SAM domain mutation associated with AEC syndrome. p63L514F keratinocytes exhibited significantly reduced proliferation compared to wild-type controls, as indicated by decreased 5-ethynyl-2′-deoxyuridine (EdU) incorporation, decreased Cyclin D1 and Cyclin D2 expression, and an increase in the cell-cycle inhibitors p21 and p27. Furthermore, p63L514F keratinocytes showed increased cell death, elevated reactive oxygen species (ROS) levels, and a decreased reduced (GSH) and oxidized (GSSG) glutathione (GSH/GSSG) ratio, indicating oxidative stress. This stress response was accompanied by a marked reduction in Solute Carrier Family 7 Member 11 (Slc7a11), a critical regulator of antioxidant defense. We further identified Slc7a11 as a likely direct transcriptional target of p63: p63 depletion reduced Slc7a11 expression, and chromatin immunoprecipitation uncovered an evolutionary conserved p63-binding enhancer upstream of the Slc7a11 promoter. Together, our findings demonstrate that p63 mutations causative of AEC syndrome impair keratinocyte proliferation, promote cell death via oxidative stress, and compromised antioxidant defenses, revealing a dual role for p63 in sustaining skin homeostasis.

## 1. Introduction

The transcription factor p63 is a master regulator of ectodermal tissue development. It is essential for the commitment of ectodermal cells to a keratinocyte fate and plays a pivotal role in epidermal development, maintenance, and differentiation [1,2]. The human *TP63* gene encodes multiple isoforms through the use of two alternative promoters and alternative splicing events, resulting in several annotated transcript variants. In the epidermis, transcription initiation starts at an internal promoter located at exon 3′, generating the most predominant ∆Np63α isoform [3]. This isoform lacks the N-terminal transactivation domain (TA) but includes a zinc finger DNA-binding domain (DBD), an oligomerization domain (OD), and all C-terminal exons, encoding a sterile-α-motif (SAM) domain and a trans-inhibitory domain (TI). The p63 SAM domain is a compact structure consisting of five helices present only in the p63α isoform [4]. In eukaryotes, the SAM domain performs multiple functions, acting as a protein–protein interaction domain as well as binding to a variety of substrates, including RNA and lipids [5,6]. The precise function of the SAM domain in the p63 protein is still unknown; however, structural studies on the p63 orthologous gene (CEP-1) in C. elegans have revealed the presence of a SAM domain that contacts the OD domain and may play a role in its stabilization [7]. Similarly, the function of the TID remains incompletely characterized, particularly in the context of ∆Np63α [3,8].

A key aspect of p63′s function lies in its ability to control gene expression through interactions with chromatin remodeling complexes. This interaction is essential for the precise orchestration of epidermal development, as p63 drives the activation of epiderma-specific genes such as basal layer epithelial keratins K5 and K14, necessary for keratinocyte identity and differentiation, while simultaneously repressing the expression of genes characteristic of simple and non-epithelial tissues, including simple epithelial keratins K8 and K18 [9,10]. This intricate control is not limited to embryonic development; in postnatal skin, p63 continues to play a vital role in maintaining the proliferative potential of progenitor cells, preserving epidermal integrity as a structural barrier, promoting cell–cell and cell–extracellular matrix adhesion, and orchestrating the complex process of epidermal differentiation [11,12].

Given these fundamental roles in epidermal biology, it is evident that the disruption of p63 function can lead to severe disorders that affect several tissues, particularly those of stratified epithelial origin.

In fact, autosomal dominant mutations in the human *TP63* gene underlie a spectrum of syndromic disorders characterized by ectodermal dysplasia, including ankyloblepharon–ectodermal defects–cleft lip/palate (AEC) syndrome [1,13]. Clinically, AEC syndrome is characterized by skin erosions, ankyloblepharon, cleft lip/palate, and ectodermal dysplasia [13,14,15]. The skin erosions, primarily affecting the scalp, neck, hands, and feet, are severe and chronic, frequently leading to infections and scarring that significantly impair patients’ quality of life [16]. Most AEC-associated mutations cluster within exons 13 and 14 of the *TP63* gene, encoding the C-terminal region of p63α [17,18]. These mutations are typically missense variants affecting the SAM domain (e.g., L514F, I537T) [19,20], while less frequently, mutations occur in the TI domain (e.g., R598L, D601V, 1709DelA, 1859DelA) [21,22]. Both types of mutations induce domain misfolding and p63 protein aggregation [21], leading to reduced transcriptional activity. In particular, the L514 amino acid localizes in the first helix of the SAM domain and is found mutated in three different amino acids (phenylalanine, valine, and serine) in AEC patients [13,18]. Using a constitutive knock-in mouse model (p63^+^/L514F) carrying a leucine-to-phenylalanine substitution at position 514 (L514F) of the p63 protein, Ferone et al. demonstrated that a mutation in the p63 SAM domain results in the downregulation of critical p63 target genes such as fibroblast growth factor receptor 2 (FGFR2) and keratin 14 (KRT14), thereby impairing the expansion of epidermal progenitor cells during development [19]. Importantly, skin fragility, a typical feature of AEC patients, is found also in p63^+^/L514F mice and is associated in both human and mouse AEC keratinocytes with an impaired regulation of the desmosome genes *Dsp*, *Dsc3*, and *Dsg1* [23], indicating that mutations in the p63 SAM domain impair both cell–cell and cell–matrix adhesion, contributing to the loss of mechanical resistance of the epidermis characteristic of AEC syndrome [22,24,25]. Unfortunately, p63^+^/L514F mice die soon after birth because of the cleft palate [19]. A new conditional knock-in mouse model was generated to enable the study of postnatal consequences of AEC mutations. This model carries the L514F mutation, with its expression mediated by Cre-recombinase under the control of a late K14 promoter [21]. Mutations in this region affect the overall structure and stability of the p63 protein by altering the packing of the helices in the SAM domain [21], thus impacting p63 function. Transcriptional activity can be restored by the insertion of amino acid variants known to reduce the intrinsic aggregation propensity of the C-terminal domain [21] or by using compounds such as doxorubicin and epirubicin that alleviate protein aggregation and restore p63 transactivation function [26].

Despite these insights, the contribution of additional p63-regulated pathways to AEC pathogenesis remains poorly understood. In this study, we investigate the role of p63 mutations in the pathogenesis of AEC syndrome, focusing on the molecular mechanisms by which p63 dysfunction affects epidermal proliferation and viability. In particular, we examine how p63 mutations induce keratinocyte cell death, providing new insights into the molecular basis of AEC syndrome.

## 2. Results

### 2.1. L514F Mutation in p63 Reduces Keratinocyte Proliferation

Previous studies have shown that both missense and frameshift mutations associated with AEC syndrome induce p63 protein aggregation, exerting a dominant-negative effect that interferes with normal p63 tetramer function [21], thus affecting the expansion and differentiation of epidermal progenitor cells during development.

To investigate the molecular alterations underlying AEC syndrome, we employed a previously established conditional knock-in mouse model harboring the AEC-associated leucine-to-phenylalanine substitution at position 514 (L514F) in the SAM domain of p63, encoded by the mouse *Trp63* gene [21]. In this model, the L514F mutation is conditionally expressed through Cre-recombinase activation under the control of the endogenous Krt14 promoter (Supplementary Scheme S1). Mice carrying the Krt14-Cre; p63L514Fflox/L514Fflox genotype (hereafter referred to as p63L514F mice) faithfully reflect the epidermal defects and skin erosions observed in AEC patients.

**Figure 1:**
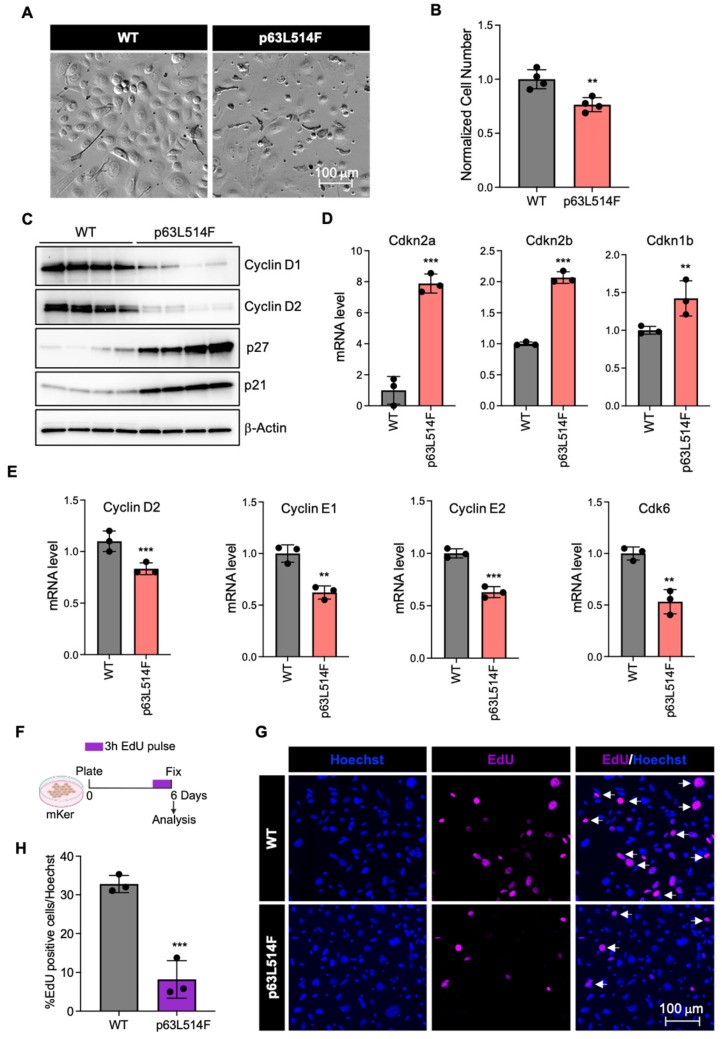
p63L514F mutation impairs keratinocyte proliferation. (**A**) Representative bright-field images of primary mouse keratinocytes isolated from WT and L514F mice and cultured for 6 days. (**B**) Total number of cells analyzed 6 days after isolation (n = 4 biological replicates/condition). (**C**) Western blot for Cyclin D1, Cyclin D2, p27, and p21 in WT and L514F keratinocytes analyzed 6 days after isolation. β-Actin was used for the normalization of protein loading (WT n = 4; L514F n = 4). (**D**,**E**) mRNA levels for cell-cycle-related genes in mouse primary keratinocytes isolated from WT and L514F 6 days after culture (n = 3 biological replicates/condition). (**F**) Scheme of the in vitro cell proliferation assay, created with BioRender.com. Mouse primary keratinocytes from WT and L514F mice were pulsed with EdU 3 h before fixation and stained 6 days after plating. (**G**) Detection by immunofluorescence analysis of proliferating EdU-positive cells (purple, indicated by the arrows) and total nuclei (Hoechst in blue). (**H**) Percentage of EdU+ nuclei/total number of nuclei in keratinocytes isolated from WT and L514F mice (n = 3 biological replicates/condition). Data are shown as mean ± SEM. Two-tailed unpaired Student’s *t*-test; ** *p* < 0.01, *** *p* < 0.005.

Primary keratinocytes were isolated from wild-type (WT) and p63L514F mice and cultured for six days, at which point WT cells reached confluence and showed active proliferation, including the shedding of cells into the medium. In contrast, keratinocytes derived from p63L514F mice exhibited a marked reduction in total cell number compared to WT controls (Figure 1A,B), indicating impaired proliferative capacity.

To assess the impact of the L514F mutation in p63 on cellular proliferation, we performed Western blot analysis on protein extract from WT and p63L514F mutant keratinocytes 6 days after culture. As shown in Figure 1C, we observed alterations in the protein expression of key cell-cycle regulators, including a notable decrease in Cyclin D1 and Cyclin D2 and an increase in the cell-cycle inhibitors p21 (Cdkn1A) and p27 (Cdkn1B) in p63L514F mutant keratinocytes. Accordingly, mRNA analysis demonstrated a significant upregulation of the cyclin-dependent kinase inhibitors Cdkn2a (p16), Cdkn2b (p15), and Cdkn1b (p27) in the mutant cells (Figure 1D). Furthermore, we observed a significant downregulation in the mRNA expression of several cell-cycle-related genes, including Cyclin D2, Cyclin E1, Cyclin E2, and Cdk6, in p63L514F mutant keratinocytes (Figure 1E).

To further validate this observation, we assessed the proliferation of WT and p63L514F keratinocytes by measuring the incorporation of the thymidine analog 5-ethynyl-2′-deoxyuridine (EdU) in newly synthesized DNA during the S-phase. Primary keratinocytes isolated from WT and p63L514F mice were cultured for 6 days, and EdU was added to the cell medium three hours before fixation (Figure 1E). Immunofluorescence analysis of Hoechst (nuclear marker) and EdU (proliferation marker) staining revealed a strong reduction in EdU-positive nuclei in p63L514F keratinocytes compared to WT controls, indicating impaired proliferation (Figure 1F). Quantification confirmed that this decrease in EdU incorporation was statistically significant, suggesting that the expression of the p63L514F protein compromises cell-cycle progression in keratinocytes (Figure 1G). These results support the notion that p63 is a key regulator of cell-cycle progression and that the AEC-associated L514F mutation impairs keratinocyte proliferation, likely through the downregulation of pro-proliferative pathways.

### 2.2. Increased Cell Death in p63L514F Keratinocytes

**Figure 2:**
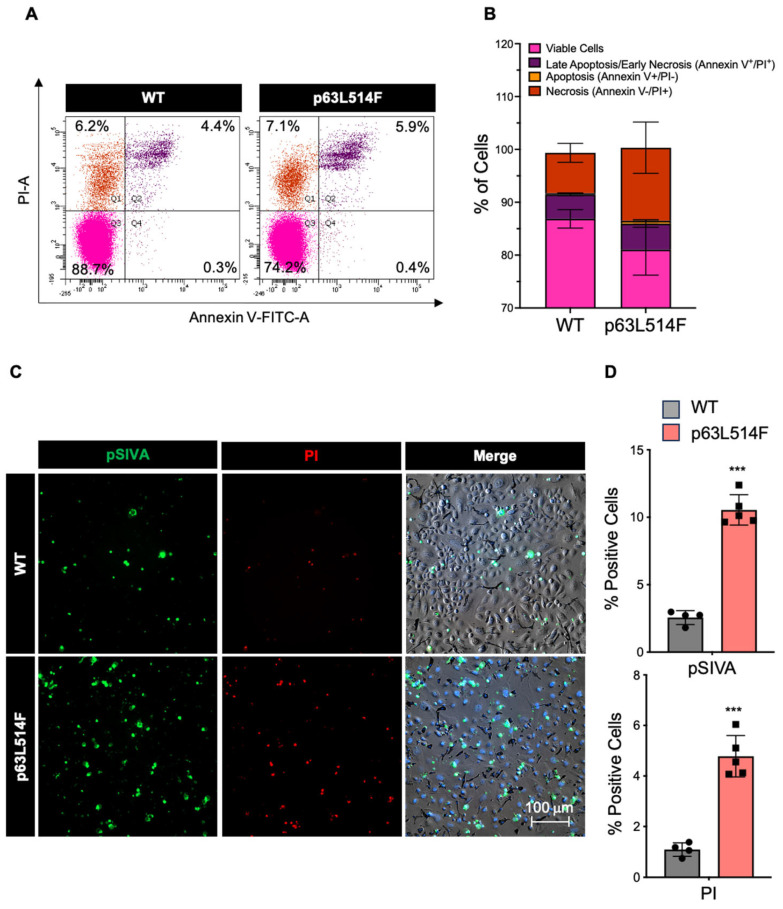
Mutation in the p63 SAM domain induces keratinocyte cell death. (**A**) Representative dot plot of flow cytometric analysis for Annexin V-FITC/PI co-staining in keratinocytes isolated from WT and L514F mice and cultured for 6 days. (**B**) Bar plot representing the percentages of viable cells (rose), late apoptotic/early necrotic cells (purple), apoptotic cells (orange), and necrotic (red) cells measured by Annexin V-FITC/PI co-staining (n = 3 biological replicates/condition). (**C**) In vitro cell death assay using pSIVA/PI staining. Mouse primary keratinocytes isolated from WT and L514F mice were pulsed with pSIVA (green) and PI (red) and analyzed by live microscopy. (**D**) Percentage of positive cells of experiment in C (n = 4 biological replicates/condition). Data are shown as mean ± SEM. Two-tailed unpaired Student’s *t*-test; (*p* > 0.05), *** *p* < 0.005.

To determine whether the proliferation defect was associated with increased apoptosis, we performed Annexin V/PI staining in WT and p63L514F keratinocytes. Flow cytometry analysis revealed a significant increase in necrotic and late apoptotic cells in p63L514F keratinocytes compared to WT controls (Figure 2A,B), indicating elevated cell death in mutant cells.

To further investigate apoptotic dynamics in real time, we employed live-cell imaging and immunofluorescence using the pSIVA (polarity-sensitive indicator of viability and apoptosis) probe, which selectively fluoresces upon binding to phosphatidylserine exposed on the outer membrane of apoptotic cells [27,28]. These assays confirmed significantly increased cell death in p63L514F keratinocytes compared to WT cells (Figure 2C,D).

**Figure 3:**
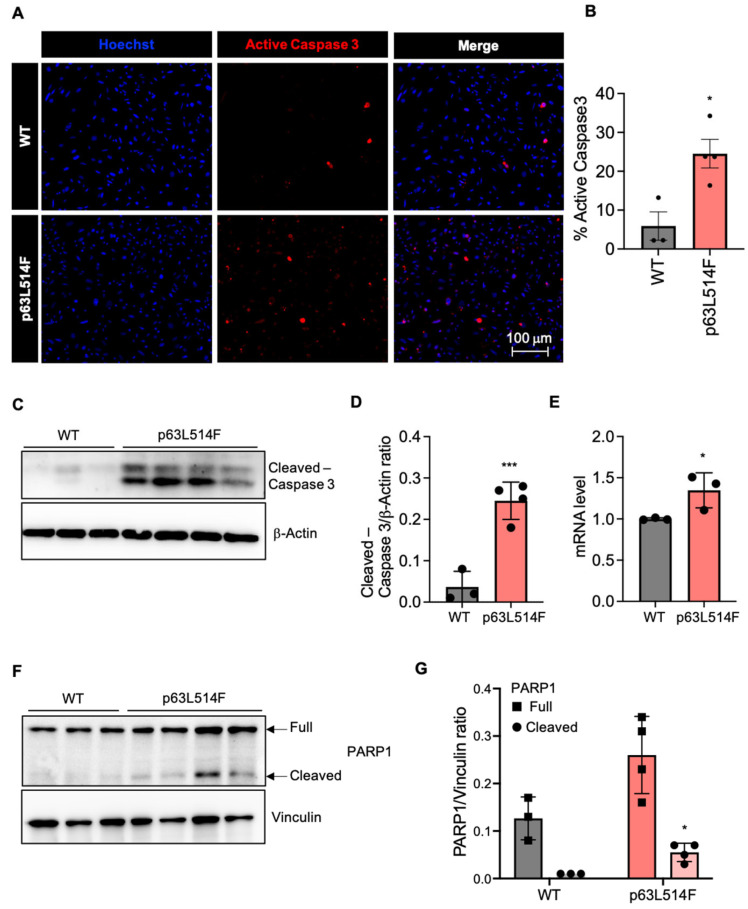
p63L514F mutation activates Caspase-3 and PARP1 cleavage. (**A**) Immunofluorescence analysis for active Caspase-3 (red) and nuclei (Hoechst, in blue) in keratinocytes isolated from WT and L514F mice and cultured for 6 days. (**B**) Percentage of active Caspase-3-positive cells from the experiment in A (WT n = 3; L514F n = 4). (**C**) Western blot for cleaved Caspase-3 in WT and L514F keratinocytes analyzed 6 days after isolation. β-Actin was used for the normalization of protein loading (WT n = 3; L514F n = 4). (**D**) Densitometric analysis of the Western blot products in C. (**E**) mRNA level of Caspase-3 in mouse primary keratinocytes isolated from WT and L514F 6 days after culture (n = 3 biological replicates/condition). (**F**) Western blot for PARP1 in WT and L514F keratinocytes analyzed 6 days after isolation. Vinculin was used for normalization of protein loading (WT n = 3; L514F n = 4). (**G**) Densitometric analysis of the Western blot products in C. Data are mean ± SEM. Two-tailed unpaired Student’s *t*-test; * *p* < 0.05 *** *p* < 0.0005.

In line with these findings, immunofluorescence analysis showed elevated levels of active Caspase-3 in p63L514F keratinocytes (Figure 3A,B). Western blot analysis further demonstrated an increase in both full-length and cleaved PARP1 protein in mutant cells compared to WT controls (Figure 3C), as confirmed by densitometric quantification (Figure 3D).

Together, these results indicate that the reduced proliferation observed in p63L514F keratinocytes is accompanied by a marked increase in cell death.

### 2.3. Increased Oxidative Stress in p63L514F Keratinocytes

During aerobic metabolism, the epidermis is continuously exposed to oxidative stress due to the generation of reactive oxygen species (ROS). To maintain redox homeostasis, p63 transcriptionally regulates key antioxidant genes, which protects proliferating keratinocytes from oxidative stress-induced apoptosis [29]. Additionally, ΔNp63α has been shown to interact with BCL-2 family members to promote cell survival, particularly in cancer cells [30]. Given the well-established role of oxidative stress in promoting apoptosis and considering the critical function of p63 in regulating cellular responses to ROS, we assessed ROS accumulation in both WT and p63L514F mutant keratinocytes, as well as in epidermal tissue isolated from WT and p63L514F mice.

**Figure 4:**
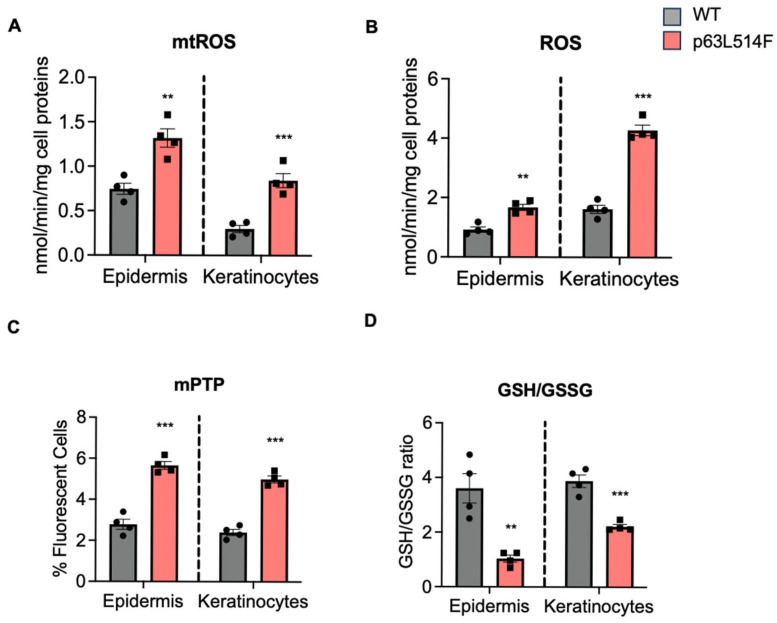
p63 mutations affect mitochondrial metabolism and oxidative–reductive balance. (**A**,**B**) Mitochondrial (mtROS; **A**) and total (**B**) ROS measured fluorometrically in technical duplicates in epidermis samples and keratinocytes from WT and mutant p63L415F mice. Data are shown as mean ± SD (n = 4 biological replicates/condition). (**C**) Fluorometric detection of the mitochondrial permeability transition pore (mPTP) opening as an indicator of mitochondrial depolarization and damage, measured in technical duplicates. Data are shown as means ± SD (n = 4 biological replicates/condition). (**D**) Ratio between reduced (GSH) and oxidized (GSSG) glutathione, measured spectrophotometrically in technical duplicates. Data are shown as means ± SD (n = 4 biological replicates/condition). Two-tailed unpaired Student’s *t*-test; ** *p* < 0.01, *** *p* < 0.005 vs. respective WT cells.

Our analysis revealed a significant increase in mitochondrial ROS (mtROS) levels in p63L514F keratinocytes and epidermis samples (Figure 4A), accompanied by elevated total ROS levels (Figure 4B), suggesting either the diffusion of ROS from mitochondria to the cytosol and/or additional cytosolic ROS production. Furthermore, mitochondria from p63L514F mice, extracted from isolated keratinocytes and total epidermis samples, exhibited a loss of membrane potential, as indicated by increased mitochondrial permeability transition pore (mPTP) opening (Figure 4C), a hallmark of mitochondrial damage typically associated with elevated mitochondrial oxidative stress and increased apoptotic susceptibility.

In addition, we detected a decreased GSH/GSSG ratio in both p63L514F keratinocytes and epidermis samples (Figure 4D), indicative of reduced antioxidant capacity. Notably, this occurred without significant changes in the expression of other major antioxidant genes (Supplementary Scheme S2A–F), further supporting the presence of oxidative stress in our AEC mutant model.

Collectively, these findings identify oxidative stress, mitochondrial dysfunction, and impaired antioxidant defenses as major contributors to the increased cell death observed in p63L514F keratinocytes.

### 2.4. Impaired Antioxidant Defense and p63 Regulation of Slc7a11 Expression in p63L514F Keratinocytes

To investigate the mechanisms underlying oxidative stress and apoptosis in p63L514F keratinocytes, we analyzed the expression of Slc7a11, a key component of the cystine–glutamate antiporter system. Slc7a11 plays a crucial role in redox homeostasis by mediating cystine uptake for glutathione synthesis, thereby limiting ROS accumulation. Previous studies have identified SLC7A11 as a direct transcriptional target of p63 in cancer cells, where it supports survival under oxidative stress conditions [30]. We thus hypothesized that Slc7a11 dysregulation could contribute to the redox imbalance observed in p63L514F mutant keratinocytes.

**Figure 5:**
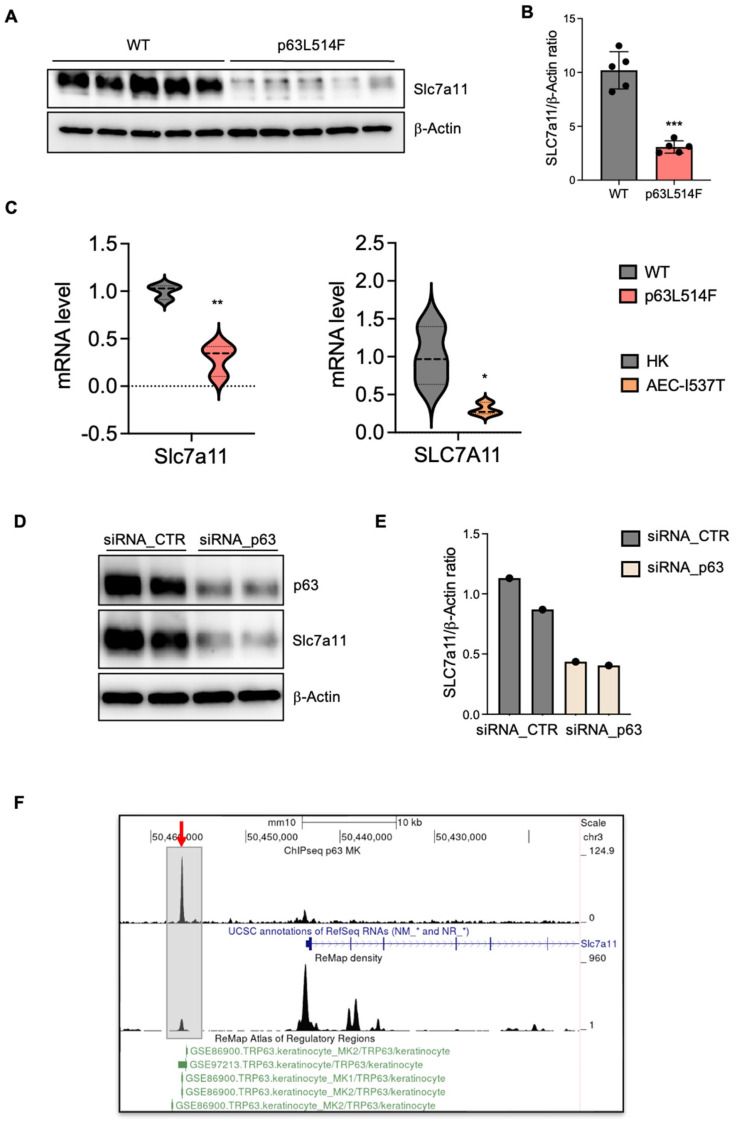
p63 regulates Slc7a11 expression. (**A**) Western blot for Slc7a11 in WT and L514F keratinocytes analyzed 6 days after isolation. α-Actin was used for the normalization of protein loading (n = 5 biological replicates/condition). (**B**) Densitometric analysis of the Western blot products in A. (**C**) mRNA level of Slc7a11 in mouse primary keratinocytes isolated from WT and L514F 6 days after culture and in human primary keratinocytes isolated from AEC patients (n = 3 biological replicates/condition). (**D**) Western blot for p63 and Slc7a11 in mouse primary keratinocytes treated with either siRNA control (siRNA_CTR) or siRNA against p63 (siRNA_p63). α-Actin was used for the normalization of protein loading (n = 2 biological replicates/condition). (**E**) Densitometric analysis of the Western blot products in D. (**F**) ChIP-seq analysis for p63 in mouse keratinocytes identified a binding site approximately 13 kb upstream of the Slc7a11 promoter (red arrow). This same region was also identified as a p63 binding site in previously published GEO datasets (green). Two-tailed unpaired Student’s *t*-test; * *p* < 0.05, ** *p* < 0.01, *** *p* < 0.005.

Western blot analysis revealed a marked reduction in Slc7a11 protein levels in p63L514F keratinocytes compared to those in WT controls (Figure 5A,B). Consistently, Slc7a11 mRNA levels were significantly decreased in mutant mouse keratinocytes (~70% reduction) and in human AEC-I537T keratinocytes, which also carry a SAM domain mutation (Figure 5C).

To confirm that this reduction was p63 dependent, we silenced p63 in WT mouse keratinocytes, which led to a notable decrease in Slc7a11 expression (Figure 5D,E). Moreover, chromatin immunoprecipitation and sequencing (ChIP-seq) experiments identified a conserved p63-binding site within an enhancer region upstream of the Slc7a11 promoter (Figure 5E), providing direct evidence that SLC7A11 is a transcriptional target of p63.

Together, these results demonstrate that p63 directly regulates Slc7a11 expression and that impaired activation of this antioxidant pathway contributes to oxidative stress and cell death, predominantly through apoptosis, although a potential contribution of other types of cell death, such as ferroptosis, cannot be excluded.

## 3. Discussion

Among the spectrum of disorders linked to p63, AEC syndrome stands out due to its severe and potentially life-threatening skin erosions. Despite the critical nature of these symptoms, there are no therapeutic approaches available for AEC syndrome. This is primarily due to the limited understanding of the intricate molecular pathways driving the disease. Our study provides novel insights into the molecular mechanisms underlying AEC syndrome by elucidating how the AEC-associated L514F mutation in p63 impairs keratinocyte proliferation and survival. We demonstrate that this mutation disrupts epidermal homeostasis through a combination of defective cell-cycle progression, increased apoptotic susceptibility, and oxidative stress dysregulation. This finding aligns with the clinical observations of AEC syndrome, where skin erosions and impaired wound healing are prominent features, potentially stemming from reduced keratinocyte proliferation.

A key finding of our study is the reduced proliferation of p63L514F keratinocytes, as evidenced by decreased Cyclin D1 and Cyclin D2 expression in mutant keratinocytes. Cyclins play a crucial role in the G1/S transition of the cell cycle, and their reduced expression suggests potential cell-cycle arrest or delay in mutant cells. Conversely, we observed increased protein levels of the cell-cycle inhibitors p27 and p21 in the mutant cells. This altered protein landscape is mirrored at the transcriptional level with the significant downregulation of several cell-cycle-promoting genes (Cyclin D2, Cyclin E1, Cyclin E2, Cdk6) and the upregulation of cyclin-dependent kinase inhibitors (Cdkn2a (p16), Cdkn2b(p15), Cdkn1b(p27)) in p63L514F keratinocytes. The consistent downregulation of multiple positive regulators and upregulation of negative regulators of cell-cycle progression provides a more comprehensive explanation for the observed significant reduction in EdU incorporation, reinforcing the conclusion that the L514F mutation profoundly disrupts the proliferative capacity of keratinocytes, likely through a multifaceted dysregulation of the cell-cycle machinery.

This is particularly interesting given that p63 is known to interact with several chromatin remodeling complexes and regulate the expression of genes involved in proliferation and differentiation [31]. Mutations located within the SAM domain might interfere with these interactions, leading to altered expression of cell-cycle regulators.

In addition to the observed proliferation defects, our results show that p63 mutations promote apoptosis in keratinocytes. The increase in necrotic and apoptotic cells in p63L514F keratinocytes, as indicated by Annexin V/PI staining and pSIVA live imaging, suggests that p63 plays a protective role against cell death. Elevated levels of active Caspase-3 and increased PARP1 cleavage further support this conclusion. These findings are in agreement with previous reports indicating that p63 can suppress apoptotic pathways in epithelial cells [32]. The observed increase in cell death by apoptosis likely exacerbates the skin erosions in AEC patients by compromising epidermal integrity and wound healing.

A novel aspect of our study is the identification of oxidative stress as a major contributor to the increased apoptosis observed in p63-mutant keratinocytes. We detected elevated levels of mitochondrial and cytosolic ROS in p63L514F keratinocytes, coupled with impaired mitochondrial membrane potential. The reduction in the GSH/GSSG ratio further supports the notion that oxidative stress is a prominent feature of p63-mutant cells. Given the known role of ROS in triggering cell death by apoptosis [33] or ferroptosis [30], our findings suggest that oxidative stress acts as a secondary driver of cell death in p63L514F keratinocytes. Our findings cannot determine whether mitochondria are the source or the targets of ROS, but they indicate that the p63 mutant phenotype is associated with mitochondrial dysfunction, which is a well-known cause of increased apoptosis. These results might provide further mechanistic insight into how p63 mutations contribute to epidermal fragility in AEC syndrome. Indeed, our study identifies SLC7A11 as transcriptional target of p63 in keratinocytes and provides evidence that p63 mutations impair the antioxidant response. The observed reduction in SLC7A11 expression at both the mRNA and protein levels in p63L514F keratinocytes and human AEC keratinocytes suggests that p63 regulates cellular redox balance through this gene. Chromatin immunoprecipitation assays further confirmed that p63 directly binds to the enhancer region of SLC7A11, establishing a direct regulatory relationship. Although we have not directly assessed the binding of the L514F mutant to the Slc7a11 enhancer in this study, a previous study by Russo et al. [21] demonstrated that AEC-associated mutations within the SAM domain, including L514F, lead to reduced binding to well-characterized p63 target gene regulatory regions. Notably, DNA pulldown experiments using in vitro translated proteins under non-aggregating conditions showed that the intrinsic DNA-binding ability of the L514F mutant is comparable to that of wild-type p63. These findings suggest that the impaired DNA binding observed in cellular contexts is not due to a defect in the DNA-binding domain itself but is more likely a consequence of aggregation induced by C-terminal mutations. Based on this, we speculate that the L514F mutant would also exhibit reduced binding to the Slc7a11 enhancer region, consistent with its aggregation-prone behavior in cells.

Given that SLC7A11 mediates cystine uptake for glutathione synthesis, its downregulation in p63-mutant cells likely contributes to the increased oxidative stress and apoptosis observed in our study. These findings align with previous studies demonstrating that p63 protects against oxidative stress-induced apoptosis in cancer cells [29].

In summary, our study identifies the p63-SLC7a11 axis as a critical mechanism in maintaining cellular redox balance and keratinocyte viability. The L514F mutation impairs this pathway, contributing to reduced proliferation, increased apoptosis, and oxidative stress in AEC syndrome.

## 4. Materials and Methods

### 4.1. Mice

K14-Cre (Krt14-CreΔneo) knock-in mice were obtained from J. Huelsken, Swiss Institute for Experimental Cancer Research, Lausanne, Switzerland, and were used to induce the expression of the p63 mutant protein. All mouse experiments were conducted at CEINGE according to the Italian ethical regulations under the under the animal license 311/2016-PR. Newborn mice at P2–P3 were used for our study.

The K14-Cre mice were crossed with p63floxL514F/floxL514F mice to generate the AEC mouse model K14-Cre;p63floxL514F/floxL514F (referred as L514F), in which the p63 mutant is expressed only in stratified epithelia. K14-Cre;p63floxL514F/floxL514F genotyping was performed by PCR using the following oligonucleotide primers:

L514F 3xFlag F: CAGCGTATCAAAGAGGAAGGAGA
L514F 3xflag R: AGCCAGAATCAGAATCAGGTGAC
Cre-recombinase F: GGCAGTAAAAACTATCCAGCAACA
Cre-recombinase R: TAACATTCTCCCACCGTCAGTA

### 4.2. Mouse Primary Keratinocyte Preparation and Cultures

Primary mouse keratinocytes were isolated from the skin of newborn mice at P3. Skin was incubated overnight at 4 °C, floating on 2 mL of a Dispase solution consisting of 0.80 U/mL Dispase II (17105-041, GIBCO, Waltham, MA, USA), 10 mM HEPES (ECM0180D, Euroclone, Pero, Italy), 0.075% sodium bicarbonate, and an antibiotic/antimycotic mixture (15240-62, GIBCO) in HBSS. On the following day, the epidermis was delicately detached from the dermis using forceps and was incubated in 1 mL of Accutase solution (ECB3056D, Euroclone, Pero, Italy) for 20 min at room temperature (RT). Subsequently, 500 μL of Trypsin–EDTA (0.25%) (25200072, GIBCO, Waltham, MA, USA) was added for 4 min at room temperature to promote enzymatic and mechanical tissue dissociation. Next, trypsin was neutralized by adding 10% fetal bovine serum (FBS) in HBSS, and the resulting cell suspension was filtered through a 70 μm cell strainer (431751, Corning, Corning, NY, USA) to eliminate undigested tissue and floating fragments.

The isolated keratinocytes were seeded into collagen-coated dishes incubated at 34 °C to promote cell adhesion. The collagen coating was composed of HBSS, BSA (100 mg/mL), HEPES (1 M, pH 6.5), and PurCol type I bovine collagen (5005-100 mL Advanced BioMatrix) and was filtered through a 0.2 μm strainer (431299, Corning, Corning, NY, USA). Keratinocytes were cultured at 34 °C and 8% CO_2_ in a low-calcium medium (LCM) (0.05 mM CaCl_2_) supplemented with 4% calcium-depleted characterized fetal bovine serum (Cha1115L, Cytivia, Marlborough, MA, USA) and epidermal growth factor (354052Corning, Corning, NY, USA).

### 4.3. Mouse Primary Keratinocyte Transfection

Mouse keratinocytes were seeded and transfected 4 days after plating with siRNA against p63 (siRNA_p63; si-G020-22061 siTOOLs BIOTECH, Munich, Germany) or Scramble (siRNA_CTR; Negative control siPOOL (NegC-120) siTOOLs BIOTECH Munich, Germany) at a 100 nM final concentration using Lipofectamine 2000 (11668027, Invitrogen, Waltham, MA, USA) in serum-free low-calcium medium (LCM), as described by the manufacturer. Briefly, pre-mixes of serum-free siRNA/LCM and serum-free Lipofectamine3000/LCM were incubated separately for 5 min at room temperature, mixed at a 1:1 ratio, and incubated for a further 20 min at room temperature. The mix was then added to the keratinocytes that were incubated for 6h in 8% CO_2_ at 34 °C. Six hours after transfection, the transfection mix was removed, and fresh supplemented LCM was added. One day after the first transfection, a second round of transfection was performed to guarantee p63 silencing in mouse keratinocytes. Cells were collected and processed for Western blot analysis 48 h after the first transfection.

### 4.4. Mouse Epidermis Isolation

This epidermal–dermal separation technique was optimized for newborn mice at P3. Phosphate-buffered saline (PBS) was warmed to 60 °C in a small beaker, while ice-cold PBS was prepared separately and kept on ice. The dorsal skin was immersed in the 60 °C PBS bath for 30 s while stirring and then quickly transferred into ice-cold PBS with stirring for 1 min. The skin was placed on Whatman paper, and using tweezers, the epidermis was gently removed and quickly frozen in liquid nitrogen for further processing. For oxido-reductive measurements, samples were homogenized using a TissueLyser II device (Qiagen, Hilden, Germany) in 1 mL PBS and filtered to obtain a single-cell suspension. A 50 µL aliquot was sonicated and used to measure protein contents. Epidermis-derived cells were aliquoted at a concentration of 100 µg proteins/0.5 mL and stored at −80 °C for the metabolic assays [34].

### 4.5. Human Primary Keratinocyte Cultures

Primary human keratinocytes (normal HK, and AEC mutant cells) (kindly provided by J. Zhou) were plated (3000 cells/cm^2^) on 80% confluent mitotically blocked 3T3-J2 cells and grown at 37 °C, 5% CO_2_ in a humidified atmosphere in KGM (keratinocyte growth medium) as described in [23]: Dulbecco’s modified Eagle’s medium (61965-026, GIBCO, Waltham, MA, USA) and Ham’s F12 medium (31765-027, GIBCO, Waltham, MA, USA) (2:1 mixture) containing characterized FBS (10%) (Cha1115L, Cytivia), 1% penicillin/streptomycin (ECB3001D, Euroclone, Pero, Italy), 4 mM L-glutamine (ECB3000D, Euroclone, Pero, Italy), 0.18 mM adenine (A2786, Sigma-Aldrich, St. Louis, MO, USA), 5 μg/mL transferrin (Sigma), 5 μg/mL insulin (12585-014, GIBCO, Waltham, MA, USA), 0.1 nM cholera toxin (C8052-5 mg, Sigma-Aldrich, St. Louis, MO, USA), 0.4 μg/mL hydrocortisone (386698-25 mg, Millipore, Burlington, MA, USA), 2 nM triiodothyronine (T6397, Sigma-Aldrich, St. Louis, MO, USA), 10 ng/mL epidermal growth factor (354052, Corning), and 10 µM ROCK inhibitor Y27632 (EN270333M005, Enzo Life Sciences, Long Island, NY, USA). When sub-confluent, cell cultures were serially propagated.

### 4.6. EdU Incorporation Assay

To assess proliferation, primary human keratinocytes were pulsed with 1 mM of EdU (ThermoFisher, Waltham, MA, USA, C10640) in keratinocyte growth medium for 3 h at 37 °C before fixation.

Cells were rinsed twice with 1X PBS and fixed with warm 4% paraformaldehyde for 10 min at room temperature. EdU was visualized with a Click-iT reaction according to manufacturer instructions.

### 4.7. SDS-PAGE and Western Blot

For SDS-PAGE, cells were lysed in LDS Sample Buffer 2X (10% glycerol, 0.01% bromophenol blue, 0.0625 M Tris-HCl pH 6.8, 3% SDS, 5% ß-mercaptoethanol) supplemented with protease and phosphatase inhibitors, ultrasonicated, boiled, and loaded on denaturing SDS-PAGE gels before Western blotting. Membranes were blocked with PBS 0.2% Tween 20 in 5% nonfat-dry milk and incubated with primary antibodies for 2 h at room temperature (RT) or overnight at 4 °C. Membranes were incubated for 1 h at RT with the appropriate horseradish peroxidase-conjugated secondary antibody and detected by chemiluminescence using the ChemiDoc Gel Imaging System. Densitometric analysis of the immunoblots was performed using Image Lab Software v.6.1.0 (Bio-Rad, Hercules, CA, USA). The antibodies used for immunoblot analysis were as follows:

Anti-p63 (EPR5701, ab124762 Abcam, Waltham, MA, USA), anti-Slc7a1 (#98051, Cell Signaling, Danvers, MA, USA) anti-Vinculin (sc73614, Santa Cruz, Dallas, Texas, USA), anti-β-Actin (sc69879, Santa Cruz, Dallas, Texas, USA), anti-cleaved Caspase-3 (Asp175) (#9661, Cell Signaling, Danvers, MA, USA), goat anti-rabbit Igg HRP-conjugate (GTXRB-003-DHRPX, ImmunoReagents Inc, Raleigh, NC, USA.), goat anti-mouse Igg HRP-conjugate (GTXMU-003-DHRPX, ImmunoReagents Inc. Raleigh, NC, USA).

### 4.8. RNA Extraction, RT-PCR, and Real-Time RT-PCR

Total RNA was extracted using a Direct-Zol micro prep kit (R2061, Zymo Research, Irvine, CA, USA). cDNA was synthesized using LunaScript RT-Supermix (E3010S, New England Biolabs, Ipswich, MA, USA). RT-qPCR to assess for mRNA relative expression performed using Luna Universal qPCR master mix (M3003L, New England Biolabs, Ipswich, MA, USA) in in an Applied Biosciences machine. Data analysis was performed using the 2^−ΔΔCT^ method [35] (Livak and Schmittgen 2001), and mRNA expression was normalized with RPLP0. Primers used in this study included the following:

SLC7A11 F: TCATCTCTCCTAAGGGCGTG
SLC7A11 R: GTTCCACCCAGACTCGTACA
RPL0 F: GACGGATTACACCTTCCCACTT
RPL0 R: GGCAGATGGATCAGCCAAGA

### 4.9. Apoptosis Detection Through Annexin V-FITC Staining

Apoptosis analyses were performed by using the Annexin V-FITC Apoptosis Detection Kit (Sigma-Aldrich, St. Louis, MO, USA, cod. APOAF) according to the manufacturer’s instructions. Stained cells were analyzed on a BD FACS Canto flow cytometer using BD FACS Diva software 8.0.3 (Duke University Flow Cytometry Shared Facility), and data were analyzed using FlowJo v10.10.

### 4.10. pSIVA Real-Time Staining

The pSIVA Real-Time Apoptosis Kit (APO004, Bio-Rad, Hercules, CA, USA) facilitates the analysis of dynamic apoptotic events in real time. To achieve this, 2 mM of CaCl2 was introduced into the low-calcium medium (LCM 4% + EGF 0.1 mg/mL) typically employed for mouse primary keratinocyte cell culture. Subsequently, the keratinocytes were washed with HBSS (Hanks’ balanced salt solution without Ca^2+^ and Mg^2+^), and three components—pSIVA (pSIVA-IANBD, 488 nm), propidium iodide (PI, 493 nm, Invitrogen), and NucBlue (NucBlue Live ReadyProbes Reagent, Hoechst 33342, Invitrogen, Waltham, MA, USA)—were added to the previously prepared medium. Acquisition was performed using the ZEISS Celldiscoverer 7 (ZEISS LSM 900).

### 4.11. Immunofluorescence

For immunostaining, cells were fixed with 4% paraformaldehyde (PFA, Electron Microscopy Sciences, Hatfield, PA, USA) in PBS for 10 min at RT. Cells were washed three times in PBS, permeabilized in 0.5% Triton X-100 (T8787, Merck, Milano, Italy) for 5 min at RT, washed again three times in PBS, and blocked with 10% goat serum (TM 16210072, GIBCO, Waltham, MA, USA). Cells were incubated with the indicated primary antibodies overnight at 4 °C in PBS with 2% goat serum. After PBS washes, the cells were incubated with secondary antibodies and Hoechst (H1399, ThermoFisher, Waltham, MA, USA) for 45 min at RT. The antibodies used for immunofluorescence analysis were cleaved Caspase-3 (D175) 5A1E (9664S, Cell Signaling, Danvers, MA, USA) and Alexa Fluor™ 594 goat anti-rabbit IgG (H+L) secondary antibody (A-11012, Invitrogen, Waltham, MA, USA).

### 4.12. Total and Mitochondrial ROS Analysis

A total of 1 × 10^6^ keratinocytes were washed with PBS and detached by gentle scraping. One 50 µL aliquot was sonicated and used to measure cellular proteins. The remaining cells, as well as epidermis-derived cells corresponding to 100 µg proteins, were treated for 30 min at 37 °C with 5µM of the ROS-sensitive fluorescent probes 5-(and-6)-chloromethyl-2′,7′-dichlorodihydro-fluorescein diacetate (CM-H2DCFDA) (ThermoFisher, Waltham, MA, USA) or with 5 µM MitoSOX (ThermoFisher, Waltham, MA, USA) to measure total and mtROS, respectively. The RFUs were converted into nanomoles ROS/mg proteins according to a titration curve performed with serial dilutions of H_2_O_2_.

### 4.13. Glutathione Measurement

A total of 1 × 10^5^ keratinocytes and epidermis-derived cells (corresponding to 100 µg proteins) were washed with 480 µL PBS, and proteins were precipitated by adding 120 µL of 6.5% *w*/*v* 5-sulfosalicylic acid. Each sample was placed on ice for 1 h and centrifuged for 15 min at 13,000× *g* (4 °C). Total glutathione was measured in 20 µL of the lysate with the following reaction mix: 20 µL stock buffer (143 mM NaH2PO4 and 63 mM EDTA, pH 7.4), 200 µL daily reagent (10 mM 5,5′dithiobis-2-nitrobenzoic acid and 2 mM NADPH in stock buffer), and 40 µL glutathione reductase (8.5 U/mL). The content of oxidized glutathione (GSSG) was obtained after the derivatization of GSH with 2-vinylpyridine (2VP): 10 µL of 2VP was added to 200 µL of lysate, and the mixture was shaken at room temperature for 1 h. Glutathione was then measured in 40 µL of sample as described. The reaction was followed kinetically for 5 min using a Synergy HT Multi-Mode Microplate Reader (Bio-Tek Instruments, Vinooski, VT, USA), measuring the absorbance at 415 nm. Each measurement was made in triplicate, and results were expressed as nanomoles glutathione/min/mg cellular proteins. For each sample, reduced glutathione (GSH) was obtained by subtracting GSSG from total glutathione.

### 4.14. Mitochondrial Extraction

Keratinocytes and epidermis-derived cells (corresponding to 100 µg proteins) were lysed in 0.5 mL mitochondria lysis buffer (50 mM Tris-HCl, 100 mM KCl, 5 mM MgCl_2_, 1.8 mM ATP, 1 mM EDTA, pH7.2) supplemented with Protease Inhibitor Cocktail III (Sigma), 1 mM phenylmethylsulfonyl fluoride, and 250 mM NaF. Samples were centrifuged sequentially at 650× *g* for 3 min at 4° C and then at 13,000× *g* for 5 min at 4 °C. The new supernatants, corresponding to the cytosolic fraction, were used for superoxide dismutase 1 (SOD1) measurement. The pelleted mitochondria-enriched fraction was washed once with lysis buffer and resuspended in 0.25 mL mitochondria resuspension buffer (250 mM sucrose, 15 mM K_2_HPO_4_, 2 mM MgCl_2_, 0.5 mM EDTA). Then, 50 µL aliquots were sonicated and used for the measurement of protein content using a BCA Protein Assay kit (Sigma Aldrich, Milan, Italy), and for quality control, 10 µg of each sonicated sample were analyzed by SDS-PAGE and immunoblotting with an anti-porin antibody (Abcam, Cambridge, UK; clone 20B12AF2) to confirm the presence of mitochondrial proteins in the extracts. The remaining 200 µL were used for the measurement of mitochondrial (mt) SOD2, as reported below.

### 4.15. Mitochondrial Permeability Transition Pore (mPTP) Opening

The opening of mPTPs, as an indicator of mitochondrial depolarization and damage, was quantified in 1 × 10^6^ keratinocytes and epidermis-derived cells by flow cytometry (Guava EasyCyte equipped with the InCyte^TM^ 3.1 software, Millipore) using the Mitochondrial Permeability Transition Pore Assay Kit (BioVision, Milpitas, CA, USA), as per the manufacturer’s instructions. The autofluorescence of unstained cells was subtracted as a blank. Results were expressed as the percentage of fluorescent cells over total cells.

### 4.16. Antioxidant Enzymes

The activity of cytosolic superoxide dismutase 1 (SOD1) and mitochondrial superoxide dismutase 2 (mtSOD2) was measured using 10 μg of cytosolic and mitochondrial proteins after the cytosol–mitochondrial separation was performed as detailed in [36] for keratinocytes and epidermis-derived cells. Samples were resuspended in 100 µL PBS and incubated with 50 μM xanthine, 5 U/mL xanthine oxidase, and 1 μg/mL oxidized cytochrome c for 5 min at 37 °C. The rate of cytochrome c reduction, which is inhibited by SOD, was monitored for 5 min by reading the absorbance at 550 nm with a Packard microplate reader EL340 (Bio-Tek Instruments, Winooski, VT, USA). Results were expressed as µmoles reduced cytochrome c/min/mg cytosolic or mitochondrial proteins. The activities of thioredoxin reductase (TrxRN1) and NAD(P)H quinone oxidoreductase 1 (NQO1) were measured with the Thioredoxin Reductase Assay Kit and the NQO1 Activity Assay Kit (both from Abcam, Cambridge, UK), as per the manufacturer’s instructions. Results were expressed as enzymatic milliunits (mU)/mg cellular proteins.

### 4.17. ChIP-Seq and Bioinformatic Analysis

For the ChIP-seq experiment, mouse keratinocytes isolated from the p0 epidermis of wild-type mice were cross-linked with 1% formaldehyde for 10 min at 37 °C, washed in PBS, resuspended in SDS lysis buffer (50 mM Tris-HCl at pH 8.1, 10 mM EDTA, 1% SDS), and sonicated (BioRuptor High Power 60 s ON 90 s OFF for 45 min on ice) in order to obtain chromatin fragment lengths of 100–1000 bp. Chromatin was diluted in ChIP dilution buffer (16.7 mM Tris-HCl at pH 8.1, 167 mM NaCl, 1.2 mM EDTA, 1.1% Triton X-100, 0.01% SDS) and incubated overnight at 4 °C with p63-specific antibodies (EPR570, Abcam) conjugated with Dynabeads**^®^** Protein A and G (ThermoFisher Scientific, Waltham, MA, USA). Immunoprecipitated samples were washed with low-salt buffer (0.1% SDS, 1% Triton X-100, 2 mM EDTA, 20 mM Tris-Hcl pH 8.1, 150 mM NaCl), high-salt buffer (0.1% SDS, 1% Triton X-100, 2 mM EDTA, 20 mM Tris-Hcl pH 8.1, 500 mM NaCl), and LiCl immune complex wash buffer (10 mM Tris-Hcl pH 8.1, 0.25 M LiCl, 1 mM EDTA, 1% Na deoxycholate, 1% NP-40) and then washed twice with TE buffer. After immunoprecipitation, DNA was eluted in 1% SDS plus 0.1 M NaHCO_3_, purified with Qiaquick columns (ThermoFisher Scientific), and quantified using a Qubit assay (Invitrogen, Waltham, MA, USA). Purified DNA was used for library construction using the KAPA Hyper prep kit from Roche (according to the manufacturer’s protocol). The prepared libraries were then sequenced on the NextSeq 500 platform. Fastq files were mapped to the *Mus musculus* genome (mm10 build) using Bowtie2 [37,38]. Peak calling from alignment results was performed using MACS2 [39,40]. Bigwig files of ChIP-seq data (GSE97213 and GSE86900) were obtained using ReMap [41].

### 4.18. Data Analysis and Statistics

Data analysis, statistics, and visualizations were performed using Prism 10 (Graphpad Software San Diego, CA, USA, www.graphpad.com). Tests are described in the figure legends.

## 5. Conclusions

In summary, our findings provide compelling evidence that the AEC-associated L514F mutation in p63 disrupts epidermal homeostasis through a multifaceted mechanism involving impaired keratinocyte proliferation, increased susceptibility to apoptosis, and a significant dysregulation of oxidative stress responses. In particular, the p63-SLC7A11 axis is a crucial pathway for maintaining keratinocyte viability and redox balance, and its disruption by mutations occurring in p63 SAM domain likely contributes significantly to the severe skin erosions and impaired epidermal integrity observed in AEC syndrome.

## Data Availability

All the data are available from the corresponding authors upon reasonable request.

## Supplementary Materials

The supporting information can be downloaded at: https://www.mdpi.com/article/10.3390/ijms26115231/s1.
