# Supplementary material for: Ankyloblepharon–Ectodermal Defects–Cleft Lip/Palate Syndrome-Linked p63 Mutations Disrupt Keratinocyte Proliferation and Survival Through Oxidative Stress and Impaired Slc7a11 Expression
Source: Int J Mol Sci. 2025 May 29;26(11):5231. doi: 10.3390/ijms26115231 (PMC12154935; doi:10.3390/ijms26115231)
Supplement: Supplementary file 1 [file ijms-26-05231-s001.zip › ijms-3615988-supplementary.pdf]

Supplementary Figures

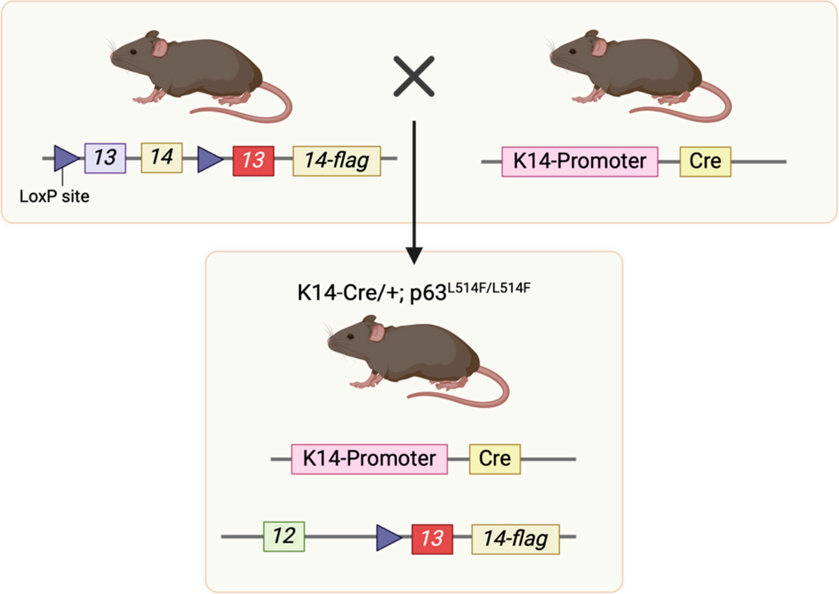

Scheme S1. Generation of the K14-Cre/+; p63L514F/L514F conditional knock-in mouse model. Schematic strategy for the generation of p63+/L514Fflox knock-in mice (Created with BioRender.com).

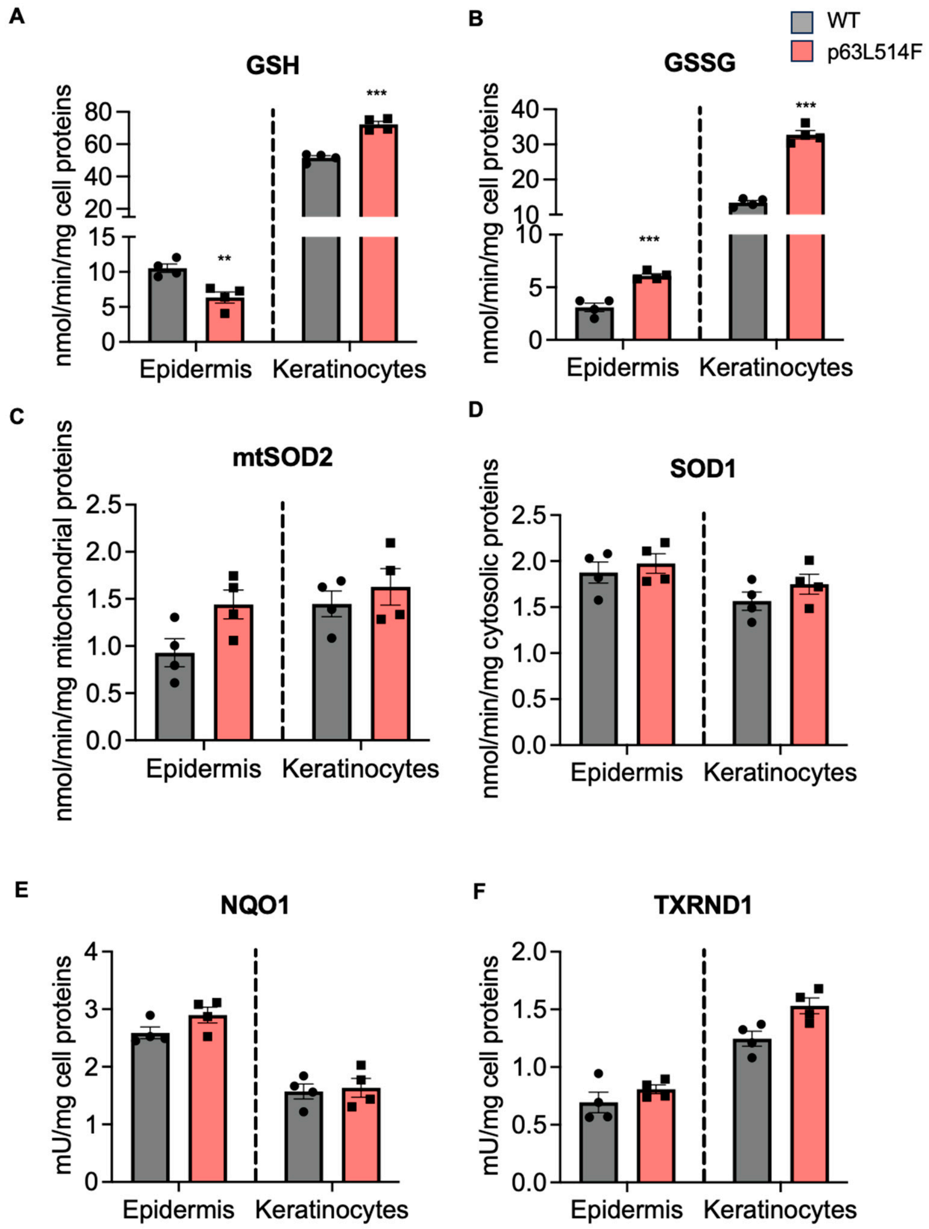

Scheme S2. Effects of p63 mutation on glutathione and antioxidant enzymes. (**A**,**B**). Reduced (GSH) and oxidized (GSSG) glutathione, measured spectrophotometrically in technical duplicates, in epidermis and keratinocytes from wild-type (WT) and mutant p63L415F mice. Data are expressed as means + SD (n = 4 biological replicates/condition). (**C**–**F**) Activity of mitochondrial superoxide dismutase 2 (mtSOD2) and cytosolic superoxide dismutase 1 (SOD1), NAD(P)H quinone oxidoreductase 1.
